# Patient’s disability and caregiver burden among Chinese family caregivers of individual living with schizophrenia: mediation effects of potentially harmful behavior, affiliate stigma, and social support

**DOI:** 10.1038/s41537-023-00418-0

**Published:** 2023-12-01

**Authors:** Dan Qiu, Yilu Li, Qiuyan Wu, Yanni An, Zixuan Tang, Shuiyuan Xiao

**Affiliations:** 1 Department of Social Medicine and Health Management, Xiangya School of Public Health, Central South University, Changsha, Hunan China; 2 Mental Health Institute, Second Xiangya Hospital, Central South University, Changsha, Hunan China

**Keywords:** Schizophrenia, Psychosis

## Abstract

Evidence on the associations between patient’s disability and caregiver burden among Chinese family caregivers of individual living with schizophrenia is lacking. This study aimed at explore the underlying mechanisms between patient’s disability and caregiver burden among Chinese family caregivers of individual living with schizophrenia. A cross-sectional study was carried out in four Chinese cities (Wuhan, Changsha, Guangzhou, and Shenzhen), between April, 2021 and March, 2022. A total of 493 patients and their family caregivers were invited to report related data. The Zarit burden interview, WHODAS 2.0, the Potentially harmful behavior scale, the Affiliate Stigma Scale, and the Multidimensional Scale of perceived social support were used to collect data. Linear regression analysis and bootstrapping analysis were conducted. The adjusted regression results showed that patients’ disability (B = 0.616; 95% CI: 0.479–0.753), potentially harmful behavior on caregivers (B = 0.474; 95% CI: 0.232–0.716), and caregiver’s low social support (B = −0.079; 95% CI: −0.158– −0.002), high level of affiliate stigma (B = 13.045; 95% CI: 10.227–15.864) were associated with higher level of caregiver burden (*p* < 0.05). In the mediation model, the direct path from patient’s disability to caregiver burden (B = 0.428, β = 0.371, *p* < 0.001) was significant and positive. Patient’s disability was indirectly associated with caregiver burden through patient’s potentially harmful behavior, caregiver’s affiliate stigma, and social support, the standardized regression coefficients ranged from 0.026-0.049 (*p* < 0.05). Patient’s potentially harmful behavior, caregiver’s affiliate stigma, and social support mediated the relationship between patients’ disability and caregiver burden. Future intervention studies designed to target these three factors may be beneficial for family caregivers of persons living with schizophrenia.

## Introduction

As a major public health problem across the world, the burden of mental disorders continues to increase, with significant impacts on health, social, and economic worldwide^1^. From 2011 to 2030, the cumulative global impact of mental disorders in terms of lost economic output will amount to $16.3 trillion^2^. Although mental disorders are significant contributors to global health burden, the global median of government health expenditure that goes to mental health is less than 2%^3^. Currently, there were 5.5 million of individuals living with schizophrenia in China. Considering the number of years lived with disability (YLDs), mental disorders in China accounted for 16.20% of all YLDs across the world^4^. In addition, the number of DALYs attributable to schizophrenia in China increased over the past 3 decades, rising by 3.03% between 2000 and 2019^4^.

Due to the scarcity of mental health resources and shortage of specialized services^5^, and the influence of traditional Chinese social culture (which emphasizes the importance of family unity and mutual care among family members), most Chinese schizophrenics are cared for by their family members^6^. Caring for individuals living with schizophrenia is challenging, families of individuals living with schizophrenia suffer from a quite heavy caregiving burden, and those family caregivers are often faced with the challenge of balancing caregiving responsibilities with their own lives^7^.

Caregiver burden was defined as “the level of multifaceted strain perceived by the caregiver from caring for a family member over time”^8^. Caring for individuals living with schizophrenia was associated with a range of psychological (such as stress), social (such as quality of life), physical (such as chronic disease), and financial problems^9–11^. Those problems, in turn, may have significant impact the quality of care, and recovery of individuals living with schizophrenia^12,13^. For example, Nuttall et al found that higher perceived family burden predicted more psychotic symptoms^13^. Considering these undesirable consequences, serval studies tried to understand predictors of family caregiving burden for individuals living with schizophrenia^14^. Researchers found that some disease-related factors including patients’ higher level of disability^15,16^, longer duration of illness^17^, more aggressive behaviors^18^ were associated higher level of family caregiving burden. In addition, some caregiver-related factors such as low social support^19^, perceived stigma^20^ were associated higher level of caregiver burden. However, very few studies focused on how those disease-related factors and caregiver-related factors will affect caregiver burden. According to Pearlin’s caregiver stress model, the behavior problems of patients with severe mental illness often give rise to some secondary stressors for family members. As a source of chronic stress for family caregivers, the behavior problems of patients may consequently lead to a greater care burden and lower quality of family care^20^. But the underlying mechanisms of the relationship between those disease-related factors, caregiver-related factors and caregiver burden among Chinese family caregivers were unclear.

The current study aimed at exploring the possible associations and underlying mechanisms between patients’ disability and caregiver burden among Chinese family caregivers of adults diagnosed with schizophrenia. Considering that there were many studies highlighting the associations between disability, aggressive behaviors of patients, social support, and affiliate stigma of caregivers, we hypothesized that: (a) disability would directly affect caregiver burden; (b) both disease-related (patients’ potentially harmful behavior) and caregiver-related stressors (social support and affiliate stigma) would mediate the association between disability and caregiver burden.

## Methods

### Ethics statement

This study was approved by the Human Research Ethics Committee of Central South University (XYGW-2021-41). Written informed consent was obtained before interviews were conducted.

### Procedure and participants

Considering the development of community-based mental health services and the economic status, four cities in southern China (including Wuhan, Changsha, Guangzhou, and Shenzhen) were selected. This was a cross-sectional study, which was carried out between April, 2021 and March, 2022. The sample size was calculated based on the formula for cross-sectional study (N = Z^2^ × (P × [1–P])/E^2^). Assuming α = 0.05 (accordingly, Z = 1.96), P (prevalence of high caregiver burden) = 40.00%^21^, and E (error)=5.00%, which means a sample size of 369 was enough for analysis. All administrative districts of the four cities were included as our sampling frame. By using random number table method, two districts were randomly selected in each city (a total of 8 districts in the four cities were selected), and 50% the community health centers in each district (63/122) were randomly selected to participated in our program, 45 of which (71.42%) were finally involved in. In the 45 included community health centers, all the eligible patients, and their caregivers (*n* = 1880) were invited to their affiliated community health centers, where trained researchers went to conduct face-to-face interviews with them. Relevant data were collected using a paper version of questionnaire that included all measurement instruments and covariates. To ensure the accuracy of the data, all staff who organized interviews had received more than one month of professional training.

Inclusion criteria of individual with schizophrenia included: (1) aged 18 or older; (2) with a clinical diagnosis of schizophrenia (diagnosis by certified psychiatrists according to ICD-10 criteria); (3) able to understand, read, and communicate with investigators in Chinese; (4) lived with family members. Patients with missing data were excluded. Inclusion criteria of family caregivers included: (1) aged 16 or older; (2) the family member is living with individual with schizophrenia and has taken the most responsibility of caregiving; (3) able to understand, read, and communicate with investigators in Chinese. Caregivers with missing data were excluded. Finally, 972 patients were enrolled. Of the 972 patients with schizophrenia approached cross 45 community health service centers, 104 patients with schizophrenia reported that they lived without any family members, which were excluded. Among 868 individuals with schizophrenia lived with family caregivers, 375 family caregivers refused to participant in the program. Finally, 493 of family caregivers gave their written informed consent and participated in the survey. Thus, 493 dyads with no missing data were included in the current analysis.

Participants who completed the questionnaire were reimbursed with RMB 20 yuan (about $ 3) in cash, as a transportation allowance.

### Caregiver burden

To assess the caregiver burden of the 493 included family caregivers, the Zarit burden interview (ZBI-22)^22^ was used. The ZBI-22 is a commonly used tool for assessing the caregiving burden in clinical and research settings (Zarit et al., 1985), in which a total of 22 items were classified into 5 dimensions: six items for relationship, seven items for emotional/well-being burden, four items for social/family burden, one item for economic burden, and four items for burden among life balance.

Participants rated the items on a 5-Likert scale with response options of never, rarely, sometimes, quite frequently and nearly always, the score of each item ranged from 0 - 4. The total scores of ZBI - 22 range from 0 to a maximum score of 88. As reported in previous studies, a higher score indicates greater caregiver burden. The Chinese version of ZBI - 22 was used in the current study, which has reported favorable validity and reliability^22,23^. In the current study, the Chinese version of ZBI - 22 indicated acceptable internal consistency, the Cronbach’s α value was 0.96.

### Disability

The 12-item WHO Disability Assessment Schedule 2.0 (WHODAS 2.0)^24^ was applied to measure participant’s level of disability, which has been previously translated into Chinese and verified for reliability and validity. The 12 questions were combined into six components, with each component assigned a score of 0-4. The six components including function for cognition, function for self-care, function for mobility, function for daily life activity, function for getting alone with others, and function for participation in the society. The disability total score is the sum of scores of the six components, ranging from 0 to 48, and a higher score indicating a higher level of disability. In this study, the WHODAS 2.0 indicated a good internal consistency, the Cronbach’s α value was 0.95.

### Affiliate stigma

The Affiliate Stigma Scale (ASS) consists of 22 items that measure the cognitive, affective, and behavioral components of affiliate stigma (Winnie et al., 2008)^25^. This scale was developed to assess affiliate stigma among caregivers of people with mental disorders (or intellectual disabilities), which has been previously translated into Mandarin and verified for reliability and validity^20^. The 22 items were combined into three domains, with each item assigned a score of 0 - 4. The three domains including cognitive-related stigma (including seven items), affect-related stigma (including seven items), and behavior-related stigma (including eight items). The total score of ASS ranging from 0 to 48, and a higher mean score of the 22 items indicates a higher level of affiliate stigma. In this study, the ASS scale indicated a good internal consistency, the Cronbach’s α value was 0.96 in the 493 included caregivers.

### Social support

The 12-item Multidimensional Scale of perceived social support (MSPSS)^26^ was applied to measure caregivers’ perceived social support from three different aspects, which including family-related support (four items), friends-related support (four items), and support came from significant others (such as colleagues, including four items). This scale was developed by Zimet in 1988. Participants rated the items on a seven Likert scale with response options of 1 = ‘very strongly disagree’ to 7 = ‘very strongly agree’, the score of each item ranged from 1 - 7. The total scores of MSPSS ranged from 12 to 84, with a higher score indicating a greater total perceived social support. In this study, the Chinese version of MSPSS scale indicated a good internal consistency, the Cronbach’s α value was 0.98.

### Potentially harmful behavior of patients

The Potentially harmful behavior scale (PHBS) was developed from the Conflict Tactics Scale (Straus, 1979), which was developed by Steinmetz in 1988. There were five indicators for psychological mistreatment and five indicators for physical mistreatment were assessed^27^. In this study, three indicators for psychological mistreatment (including screaming and yelling, threatening with physical force, and verbal abuse, such as using a harsh tone of voice, insulting, calling names, and swearing) and four indicators for physical mistreatment (including hitting or slapping, shaking, handling roughly in other ways, feeling afraid that patient might hit or try to hurt him/her) were used to assess patient’s potentially harmful behavior (PHB). Specifically, caregivers were asked to rate how often (0 = never and 4 = all the time) they have experienced the behavior by patients they were caring for. Responses were summed to create a measure of PHB, with a higher score representing a more frequent PHB of patients. In this study, the Chinese version of PHBS scale indicated a good internal consistency, the Cronbach’s α value was 0.96.

### Covariates

Sociodemographic variables of patients including gender, age, duration of disease, education level, marital status (married/single/divorced/widowed), work status, were evaluated. See Table 3 for the details. For caregivers, sociodemographic variables including gender, age, duration of caring, education level, marital status (married/single/divorced/widowed), long-term physical conditions (yes or no), work status, were evaluated. See Table 3 for the details.

### Statistical analyses

Data analysis was conducted by using SPSS 26.0 and AMOS 23.0. The differences between caregiver burden of Chinese family caregivers were examined using the single factor t test and variance analysis. Regression coefficient and 95% confidence intervals were calculated to examine the associations between patient’s disability and caregiver burden using linear regression analysis^28^. For linear regression, the total score of caregiver burden was used as the dependent variable^29^. In the adjusted regression models, known and potential confounders were controlled. Specifically, Model 1 was unadjusted model, Model 2 adjusted for caregiver’s age, education level, work status, kinship, caregiving duration, and patients’ gender, onset duration, marital status, and work status. All p-values refer to two-tailed tests and a *p* value < 0.05 was considered as statistically significant.

Pearson’s correlation analysis was conducted to examine the correlations between patient’s disability, potentially harmful behavior, caregiver’s perceived affiliate stigma, social support, and caregiver burden. The mediation effects analysis was conducted by using AMOS 23.0. The measurement model for caregiver burden was tested by using confirmatory factor analysis (CFA), which examines the goodness of fit of the underlying constructs in our proposed mediation model. The hypothesized directionality of the relationships among the constructs and overall fit of the mediation model were examined by using the path analysis. To test how well the model fitted the included data, the results of χ^2^ test, the root mean square error of approximation (RMSEA), the comparative fit index (CFI), and the non-normed fit index (NNFI) were reported. An RMSEA value smaller than 0.08, both CFI and NNFI values > 0.85 mean a reasonable model fit^30^. To identify the mediation effects of patient’s potentially harmful behavior, caregiver’s perceived affiliate stigma, and social support for the associations between patient’s disability and caregiver burden, bootstrap analysis (bias-corrected CIs based on 5000 resamples) was conducted^31^. A statistically significant mediation effect is observed when the 95% CI did not include zero^32^. The proportion mediated (PM) was calculated to evaluate the effect size of different paths in the mediation analysis for patient’s disability and caregiver burden^30^.

## Results

### Descriptive statistics

With completing the measurement of patient’s disability and potentially harmful behavior, caregiver’s affiliate stigma, social support, caregiver burden and related covariates as one of the inclusion criteria, 493 patients with schizophrenia and their caregivers were available for the final analysis.

Specially, the mean age of individuals living with schizophrenia was 46.59 years (S.D. = 13.99), most of them were older than 50 years old (58.77%), and 53.14% of the patients with schizophrenia were female. About three quarters of the patients (74.04%) had an onset duration more than 10 years, 46.86% were currently married or cohabitating with someone.

Of the included 493 caregivers, the mean age of them was 57.26 years (S.D. = 12.95), most of them were older than 40 years old (65.71%), and 49.09% of the caregivers were female. About three-quarters of the caregivers (77.08%) reported a caregiving duration more than 10 years, 86.62% were currently married or cohabitating with someone, 39.96% received primary education, and 31.03% had a full-time job (Table 1).

**Table 1:** Characteristics of the participants by caregiver burden (*n* = 493).

**Caregiver-related factors**				
**Variables**	***n*/%**	**Caregiver burden (Mean/SD)**	***t*/*F***	***p***
**Gender**			−0.033	0.973
Male	251 (50.91)	37.45 ± 22.89		
Female	242 (49.09)	37.52 ± 24.13		
**Age (year)**			6.481	<0.001
18–39	64 (12.98)	27.48 ± 25.20		
40–49	55 (11.15)	32.27 ± 24.05		
50–59	149 (30.23)	39.08 ± 23.29		
≥60	225 (45.64)	40.54 ± 22.10		
**Marital status**			2.469	0.086
Single	23 (4.66)	33.08 ± 30.66		
Married	427 (86.62)	37.00 ± 23.12		
Divorced/Widowed	43 (8.72)	44.58 ± 22.00		
**Education level**			3.707	0.012
Primary school or below	197 (39.96)	40.82 ± 23.31		
Middle school	165 (33.47)	37.17 ± 23.65		
High school	85 (17.24)	34.88 ± 22.64		
University or above	46 (9.33)	29.08 ± 23.09		
**Work status**			3.685	0.012
Full-time job	153 (31.03)	35.28 ± 24.58		
Part-time job	28 (5.68)	28.64 ± 20.78		
Unemployed	186 (37.73)	41.43 ± 23.46		
Retired	126 (25.56)	36.29 ± 21.91		
**Kinship**			6.110	<0.001
Parent	230 (46.65)	40.56 ± 22.72		
Spouse	154 (31.24)	37.66 ± 23.44		
Child	45 (9.13)	24.84 ± 22.99		
Siblings or other	64 (12.98)	34.48 ± 23.89		
**Caregiving duration (year)**			11.542	<0.001
0–9	113 (22.92)	29.00 ± 22.45		
10–19	123 (24.95)	34.47 ± 22.60		
20–29	136 (27.59)	40.14 ± 22.61		
≥30	121 (24.54)	45.47 ± 23.39		
**Chronic disease**			0.215	0.643
No	319 (64.71)	37.12 ± 23.69		
Yes	174 (35.29)	38.14 ± 23.16		
**Patient-related factors**				
**Gender**			10.261	<0.001
Male	231 (46.86)	41.06 ± 23.15		
Female	262 (53.14)	34.33 ± 23.37		
**Age (year)**			0.959	0.430
18–29	60 (12.17)	38.00 ± 24.51		
30–39	109 (22.11)	41.18 ± 21.96		
40–49	115 (23.33)	35.94 ± 23.93		
50–59	120 (24.34)	35.97 ± 24.45		
≥60	89 (18.05)	36.62 ± 22.67		
**Onset duration (year)**			9.301	0.010
1–10	128 (25.96)	30.85 ± 22.63		
11–20	180 (36.51)	40.51 ± 23.83		
>20	185 (37.53)	39.12 ± 22.79		
**Marital status**			6.053	0.003
Single	206 (41.78)	41.62 ± 22.71		
Married	231 (46.86)	33.87 ± 23.30		
Divorced/Widowed	56 (11.36)	37.12 ± 24.94		
**Education level**			1.511	0.222
Primary school or below	178 (36.11)	35.80 ± 24.47		
Middle/High school	273 (55.38)	39.08 ± 22.65		
University or above	42 (8.51)	34.16 ± 24.23		
**Work status**			11.164	0.011
Full-time job	46 (9.33)	24.50 ± 21.97		
Part-time job	18 (3.65)	25.72 ± 23.78		
Unemployed	391 (79.31)	39.51 ± 23.20		
Retired	38 (7.71)	37.86 ± 22.19		

### Associations between socio-demographics, PHB, stigma, social support and caregiver burden

As presented in Table 1, caregiver with older age, lower education level, have no job, were parents of patients, with longer caregiving duration were significantly associated with higher level of caregiver burden (*p* < 0.05). Also, patients who were male, with longer onset duration, were single, have no job, were associated with higher level of caregiver burden (*p* < 0.05). These demographic variables that significantly associated with the dependent variables were included in the adjusted models.

The adjusted regression results showed that patients’ higher level of disability (B = 0.616; 95% CI: 0.479–0.753), PHB on caregivers (B = 0.474; 95% CI: 0.232–0.716), and caregiver’s low social support (B = −0.079; 95% CI: −0.158–−0.002), higher level of affiliate stigma (B = 13.045; 95% CI: 10.227–15.864) were associated with higher level of caregiver burden (*p* < 0.05) (Table 2). See Table 2 for the details.

**Table 2:** Linear regression analysis of related variables on caregiver burden.

Variables	Model 1^a^			Model 2^b^		
	B	95% CI	*P*	B	95% CI	*P*
**Social function**	0.676	0.547-0.804	<0.05	0.616	0.479-0.753	<0.05
**Patient’s PHB on caregiver**	0.536	0.298-0.744	<0.001	0.474	0.232-0.716	<0.001
**Stigma**	13.071	10.310-15.830	<0.001	13.045	10.227-15.864	<0.001
**Social support score**	−0.102	−0.180- −0.023	<0.001	−0.079	−0.158- −0.002	<0.05

^*^p < 0.05.

^a^Model 1 was unadjusted model; ^b^Model 2 adjusted for caregiver’s age, education level, work status, kinship, caregiving duration, and patients’ gender, onset duration, marital status, and work status.

### Correlations among the variables

The Pearson’s correlation analysis (Table 3) showed that patient’s disability was positively correlated with patient’s PHB on caregivers, caregiver’s affiliate stigma, caregiver’s social support, and caregiver burden (r = 0.322, 0.254, −0.298, and 0.539), respectively. Patient’s PHB on caregiver was significantly correlated with caregiver’s affiliate stigma (r = 0.287), caregiver’s social support (r = −0.162), and caregiver burden (r = 0.391), respectively. Caregiver’s perceived affiliate stigma was also significantly correlated with caregiver’s social support (r = −0.349) and caregiver burden (r = 0.512). Caregiver’s social support was significantly correlated with caregiver burden (r = −0.348).

**Table 3:** Results of Pearson’s correlation between different variables.

Number	Variables	Mean ± SD	1	2	3	4
**1**	Patient’s disability	27.48 ± 13.00	1			
**2**	Patient’s PHB on caregiver	3.81 ± 6.91	0.322^**^	1		
**3**	Caregiver’s affiliated stigma	2.21 ± 0.61	0.254^**^	0.287^**^	1	
**4**	Caregiver’s social support	41.97 ± 21.28	−0.298^**^	−0.162^**^	−0.349^**^	1
**5**	Caregiver burden	37.48 ± 13.00	0.539^**^	0.391^**^	0.512^**^	−0.348^**^

**p < 0.05.

### Results of model testing, path coefficients, and mediation effects

**Fig. 1:**
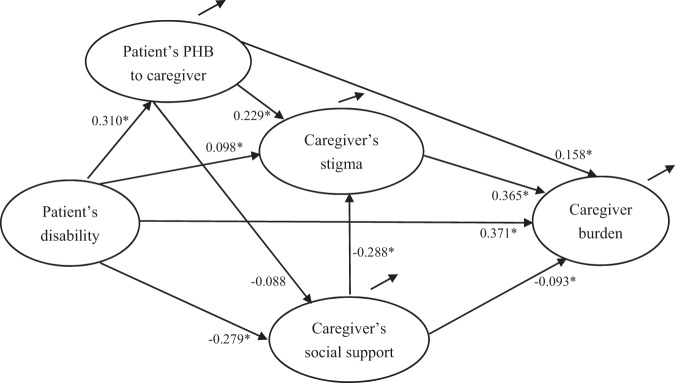
The proposed mediation model with standardized coefficients (β value). *p < 0.05. For simplicity reasons, the demographic variables controlled in the model were not presented in the figure.

Verification analysis of the structural equation model proved that the model fit the data well (Fig. 1), χ^2^(109) = 351.747, *p* < 0.001, CFI = 0.979, NNFI = 0.973, RMSEA = 0.06. All factor loadings were significant at *p* < 0.001. See Fig. 1 for the details.

As hypothesized, the direct path from patient’s disability to caregiver burden (β = 0.371, *p* < 0.001) was significant and positive. Higher level of disability was associated with increased frequency of potentially harmful behavior (β = 0.310, *p* < 0.001), affiliate stigma (β = 0.098, *p* < 0.05), and lower level of social support (β = −0.279, *p* < 0.001). Higher level of potentially harmful behavior was associated with higher level of affiliate stigma (β = 0.229, *p* < 0.001), and caregiver burden (β = 0.158, *p* < 0.001). Lower level of social support was associated with higher level of affiliate stigma (β = −0.288, *p* < 0.001), and caregiver burden (β = −0.093, *p* < 0.05). Also, higher level of affiliate stigma was associated with higher level of caregiver burden (β = 0.368, *p* < 0.001). The association between potentially harmful behavior and social support was not significant (*p* > 0.05).

Bootstrapping analyses indicated that patient’s disability was indirectly associated with caregiver burden through patient’s PHM to caregiver (β = 0.049, *p* < 0.01, PM = 9.12%). Further analyses showed that patient’s disability was indirectly associated with caregiver burden through patient’s PHM to caregiver and caregiver’s affiliate stigma (β = 0.026, *p* < 0.01, PM = 4.84%). Also, patient’s disability was indirectly associated with caregiver burden through caregiver’s affiliate stigma (β = 0.036, *p* < 0.05, PM = 6.70%), and further analyses showed that patient’s disability was indirectly associated with caregiver burden through caregiver’s affiliate stigma and caregiver’s social support (β = 0.029, *p* < 0.01, PM = 5.40%. Lastly, patient’s disability was indirectly associated with caregiver burden through caregiver’s social support (β = 0.026, *p* < 0.05, PM = 4.84%). The mediation effects of patient’s PHM to caregiver and caregiver’s social support for the associations between patient’s disability and caregiver burden was not significant (*p* > 0.05). See Table 4 for the details.

**Table 4:** Bootstrap analyses of total, direct, indirect effects of the mediation model.

Paths	β	Boot *SE*	95%*CI*^a^	*p*
**Direct effect**				
Patient’s disability → caregiver burden	0.371	0.009	0.292-0.450	*p* < 0.01
**Indirect effect**				
Patient’s disability → patient’s PHB to caregiver → caregiver burden	0.049	0.013	0.024–0.076	*p* < 0.01
Patient’s disability → patient’s PHB to caregiver → caregiver’s affiliate stigma → caregiver burden	0.026	0.007	0.015–0.040	*p* < 0.01
Patient’s disability → patient’s PHB to caregiver → caregiver’s social support → caregiver burden	0.003	0.006	0.000–0.007	*P* = 0.06
Patient’s disability → caregiver’s affiliate stigma → caregiver burden	0.036	0.017	0.003–0.068	*p* < 0.05
Patient’s disability → caregiver’s social support → caregiver burden	0.026	0.012	0.004–0.051	*p* < 0.05
Patient’s disability → caregiver’s social support → caregiver’s affiliate stigma → caregiver burden	0.029	0.008	0.016–0.046	*p* < 0.01
**Total effect**	0.537	0.036	0.469–0.610	*p* < 0.01

^a^Percentile method was presented based on 5000 bootstraps samples.

## Discussion

### Key findings

This study provided evidence that patients’ disability was associated with higher level of caregiver burden, and patient’s potentially harmful behavior, caregiver’s affiliate stigma, and social support mediated the relationship between patients’ disability and caregiver burden. Patient’s disability was indirectly associated with caregiver burden through 5 paths, the standardized regression coefficients, β value ranged from 0.026 to 0.049 (*p* < 0.05). The mediation effects of patient’s PHM to caregiver and caregiver’s social support for the associations between patient’s disability and caregiver burden was not significant (*p* > 0.05).

### Mediation effects of patient’s PHB

Globally, few studies have explored the associations between disability of patients with schizophrenia and care burden among their family caregivers, the evidence is limited. Kumar et al. found in their study that caregiver burden is associated with disability in schizophrenia^15^, with a higher level of disability was associated with higher level of caregiver burden. The findings in this study demonstrate that patient’s disability had a direct effect on caregiver burden of Chinese family caregivers, which was consistent with previous findings.

Refer to the indirect effect of patient’s disability on caregiver burden of Chinese family caregivers, we found that patient’s disability could impact patient’s potentially harmful behavior to caregiver and further increase caregiver burden. Several studies have indicated a high prevalence of violent behavior among inpatients with mental disorder, explored the associations between severity of their disease and prevalence of violent behavior^33,34^. There is little awareness of family violence by outpatients with severe mental illness, and few studies have addressed this issue^33^. It is said that family caregivers were most likely to be the targets of patient-perpetrated violence^35^, which constitutes a particular burden for caregivers, yet remains largely overlooked^18^. This study provides some new evidence, indicated that the impact of potentially harmful behaviors of patients (including potentially physical, verbal, and psychological violence/abuse) on the associations between patients’ disability and caregiver burden, which was consistent with previous opinions^18^. Considering that family violence of patients with severe mental illness may lead to violence-related negative health outcomes in family caregivers (such as suicide), we believe more research is needed in the future, which will contributed to decrease the burden of severe mental illness.

### Mediation effects of caregiver’s affiliate stigma

Individuals who have disabilities and their families are vulnerable to daily acts of discriminatory treatment that continue throughout their daily lives, which may then increase caregivers’ perceived stigma^36,37^. According to Pearlin’s caregiver stress model in 1989, the behavior problems of patients with severe mental illness often give rise to some secondary stressors for family members, such as stigmatizing interactions. As a source of chronic stress for family caregivers, the stigmatization of mental illness in family caregivers can lead to physical and mental health problems, such as cardiovascular health issues, depression, burn out, and low life satisfaction^20,37^. These negative health outcomes, in turn, may consequently lead to a greater care burden and lower quality of family care^20^. In this study, patient’s disability could increase the level of caregiver’s perceived stigma and further increase caregiver burden. Furthermore, patient’s disability could impact patient’s potentially harmful behavior, then increase the level of caregivers’ affiliate stigma and further increase caregiver burden. Thus, anti-stigma interventions to reduce stigma’s negative impact among Chinese family caregivers of people with severe mental illness are key for the future^38^.

### Mediation effects of caregiver’s social support

We also found that patient’s disability could decrease the level of caregiver’s social support and further increase caregiver burden. Social support plays an important role in reducing not only physical burden, but also psychological burden in the family caregivers^39^. Several studies have summarized related determinants of family caregiving burden, they found that the dependency level of the care recipient is the most important predictor of care burden, and the dependency level of the care recipient was reported to associated with the severity of illness^40^. At the same time, social support acts as a protector between care stressors and caregiving burdens. However, caregiver social support is often negatively affected by the severity of the patient’s illness. This refers to the fact that as the severity of the disease increases, patients and their families may feel more social isolation, poorer family functioning, and the ability and opportunity to seek social support may be negatively affected^7,40–42^.

To sum up, the findings in our path analysis further confirms the negative impact of patient’s condition on caregivers’ social support and caregiving burden. And, the relationship between low social support and high caregiver burden and their significant effect on the disease course indicates a vicious cycle that affects patients living with schizophrenia, their family caregivers, and the clinical course of the disease^13,40^. The mediation effect of social support also indicates that when these family caregivers are provided sufficient social support to meet their needs, perceived affiliate stigma of caregivers may be correspondingly decreased, which in turn leads to a reduction in the burden of care^43^.

### Future implications

As one of the countries with high burden of mental disorders^44^, the Chinese government was eager to establish a community-based comprehensive mental health system as early as 20 years ago, emphasizing the urgent need to improve community-based mental health services^45,46^. Although the prevalence of severe mental illness is high in China, the Chinese health system resources have not yet sufficiently responded to the burden of severe mental illness^46^. Family caregivers still play a crucial role in mental health care in China. Considering the high level of caregiver burden reported by family caregivers in this study, the Chinese health system should take effective measures, improve the availability of community-based mental health services and mental health resources, reducing the burden of care and ultimately contributing to improved social functioning of patients^47,48^.

Currently, an important goal in the field of global mental health is to develop evidence-based interventions that target not only the individual living with schizophrenia, but also for the whole family^49^. Based on our findings, we believe there is a particular need for effective interventions to improve the occupational skills and functioning of patients and caregivers in daily household tasks, and to further assist family caregivers in organizing these tasks to promote patient engagement^14,50^. Influenced by traditional Chinese culture and social environment, family members in China usually feel shame and deal with the stigmatization of mental illness by hiding or withdrawing as a coping strategy. To avoid stigmatization, some families even segregate mentally ill persons. Therefore, in Chinese culture, taking measures against family members is important in anti-stigmatization efforts. Currently, law and policy are essential but insufficient to end stigma, improve social support in mental health. Relevant departments should issue more relevant policies and support more intervention projects. Lastly, future studies should further investigate the effect of coping styles of family members on the associations between patient’s disability and care burden, and take individualized, targeted measures for different population.

### Limitations

Firstly, given the cross-sectional nature of this study, it can’t be used to analyze the cause and effect. Although we conducted mediation analysis, future longitudinal research to detect the impact of changes of patient’s potentially harmful behavior, caregivers’ social support, affiliate stigma on caregiver burden is warranted. To ensure the study was accessible to participants with a range of difficulties, travel expenses were paid (¥20). Attempts were made to include participants who are hospitalized. For individuals in the community, participants are personally invited through telephone invitations. Despite these efforts, some patients refused to participate in the program, which may have affected the results.

## Conclusion

Patient’s potentially harmful behavior, caregiver’s perceived affiliate stigma, and social support mediated the relationship between patients’ disability and caregiver burden. Future intervention studies which be designed aiming at the three factors may be beneficial for family caregivers of persons living with schizophrenia. Firstly, there is a particular need for effective interventions to improve the occupational skills and functioning of both patients and caregivers in daily household tasks. Furthermore, interventions to assist family caregivers in organizing these caregiving tasks and promote patient engagement is also crucial for reduce the burden of care. Lastly, taking measures targeting family members are important in the fight against the stigmatization of serious mental disorders, which could help to reducing the burden of care.

## Data Availability

The data that support the findings of this study will be shared after approval of a proposal and with a signed data access agreement.
